# Left ventricular diastolic function in patients with type 2 diabetes treated with a dipeptidyl peptidase-4 inhibitor- a pilot study

**DOI:** 10.1186/1758-5996-6-103

**Published:** 2014-09-24

**Authors:** Katia Camarano Nogueira, Meive Furtado, Rosa Tsuneshiro Fukui, Marcia Regina Silva Correia, Rosa Ferreira dos Santos, José Lázaro Andrade, Maria Elizabeth Rossi da Silva

**Affiliations:** Laboratório de Carboidratos e Radioimunensaio LIM-18 do Hospital das Clínicas da Faculdade de Medicina da Universidade de São Paulo, Av Dr Arnaldo, 455 sala 3324, Sao Paulo, SP Brazil; Serviço de Ecocardiografia do Instituto de Radiologia do Hospital das Clínicas da Faculdade de Medicina da Universidade de São Paulo, Sao Paulo, Brazil

**Keywords:** Type 2 diabetes mellitus (T2DM), Dypeptidil-peptidase-4 inhibitor, Bedtime NPH insulin, Cardiovascular function, Glucagon-like peptide 1

## Abstract

**Background:**

Blood glucose control is fundamental albeit not enough to prevent diabetic macrovascular complications. Dipeptidyl peptidase-4 (DPP-4) inhibitors are effective in improving metabolic parameters in patients with type 2 diabetes mellitus (T2DM) but little is known about its cardiovascular effects. We compared the DPP-4 inhibitor sitagliptin with bedtime NPH insulin (NPH) as add-on therapy in patients with T2DM, aiming to ascertain which drug would have additional cardioprotective effects.

**Methods:**

Thirty-five T2DM patients inadequately controlled with metformin plus glyburide were randomized to receive sitagliptin (n = 18) or NPH (n = 17) for 24 weeks. Fasting plasma glucose, HbA1c, lipid profile, C-reactive protein, active glucagon-like peptide (aGLP-1) levels, 24-hour ambulatory blood pressure measurement and comprehensive 2-dimensional echocardiogram were determined before and after treatments.

**Results:**

Both sitagliptin and NPH therapies decreased HbA1c levels after 24 weeks. Fasting plasma glucose and triglyceride levels decreased in the NPH group whereas only sitagliptin increased aGLP-1 levels. Left ventricular diastolic dysfunction (LVDD) was detected in 58.6% of twenty-nine patients evaluated. Beneficial effects in LVDD were observed in 75% and 11% of patients treated with sitagliptin and NPH, respectively (*p* = 0.015). Neither therapy changed C-reactive protein or blood pressure.

**Conclusions:**

Sitagliptin and bedtime NPH were similarly effective on glucose control. Improvement in LVDD in T2DM patients treated with sitagliptin was suggested, probably related to the increase of aGLP-1 levels. Therefore, DPP-4 inhibitor seems to have cardioprotective effects independent of glucose control and may have a role in the prevention of diabetic cardiomyopathy.

## Background

Beta-cell dysfunction, insulin resistance and impaired suppression of glucagon are key pathologic defects in type 2 diabetes (T2DM), partially dependent on the reduced incretin effect [1, 2].

In order to achieve appropriate control of diabetes, address those dysfunctions and prevent vascular complications, the majority of patients require multiple oral therapies usually followed by insulin in the long-term. Bedtime NPH insulin has been well established as third line agent in T2DM treatment [1, 3].

Recently, incretin-based therapy with glucagon-like peptide 1 (GLP-1) analogues or dipeptidyl peptidase-4 (DPP-4) inhibitors, that increase GLP-1 levels, has been proposed as an alternative to currently available anti-hyperglycemic agents. GLP-1 acts through GLP-1 receptors expressed not only in α-, β-, δ-cells of the pancreatic islets, but in a wide range of tissues including the human heart [2].

Besides glucose control, DPP-4 inhibitors improve several cardiovascular risk factors. They may lower blood pressure, improve renal and endothelial dysfunction and lipid levels and reduce inflammatory markers, oxidative stress and platelet aggregation in patients with T2DM. In addition, are weight neutral and rarely cause hypoglycemia [2, 4].

Furthermore, positive effects on the myocardium have been described in ischemic heart disease. Experimental evidence from animals and patients (without diabetes) have revealed inotropic and vasodilator effects of native GLP-1 and its analogues, increase in myocardial glucose uptake and reduction in infarct size when given either prior to injury or at the point of reperfusion [5–7]. The DPP-4 inhibitor sitagliptin in animals and in nondiabetic subjects have shown similar results [4, 8, 9], suggesting a potential cardioprotective effect. DPP-4 inhibition may also increase the concentration of many peptides with potential vasoactive and cardioprotetctive effects [8].

Only recently, studies have attested that DPP-4 inhibitors are safe in T2D patients and may possibly decrease the risk of adverse cardiovascular events [10, 11].

However, definite relationship between DPP-4 inhibitors and better cardiovascular outcomes beyond metabolic control remains to be proven and no study analyzed the effect of these drugs on myocardial function in patients with T2DM in a non-ischemic condition. Therefore, we conducted a comparison of sitagliptin with bedtime NPH insulin as add-on therapy in T2DM patients inadequately controlled with metformin plus glyburide, aiming to ascertain whether the DPP-4 inhibitor would have additional cardioprotective effects.

## Methods

Inclusion criteria: 35 Outpatients with T2D (20 F/15 M) aged 57 ± 7 years (mean ± SD) inadequately controlled with metformin plus glyburide were randomized to receive sitagliptin 100 mg once daily (SITA group) or bedtime NPH insulin- final dose: 11.0 ± 6.7 IU (NPH group) (Table 1). Exclusion criteria included: severe heart failure, respiratory failure, uncontrolled hypertension, coronary heart disease, arrhythmias, hepatic and renal dysfunctions, endocrine and gastrointestinal disorders, malignancy, alcohol abuse, use of insulin, beta blockers or calcium channel antagonists and type 1 DM.

**Table 1:** **Clinical and biochemical characteristics of 35 patients with type 2 diabetes at baseline and after 24-wk of sitagliptin or bedtime NPH insulin therapy**

	SITA	SITA	NPH	NPH
	Baseline	After 24-wk	Baseline	After 24-wk
Number of patients	18		17	
Female/Male	09/09		11/06	
Age (years)	55.1 ± 6.7		58.4 ± 6.9	
Diabetes duration (years)	10.9 ± 5.8		10.9 ± 7.5	
Weight (kg)	69.4 ± 12.0	69.9 ± 11.4	73.1 ± 10.1	74.0 ± 10.4
BMI (kg/m^2^)	26.5 ± 2.7	26.8 ± 2.6	27.5 ± 2.5	27.8 ± 2.8
Waist-hip ratio	0.97 ± 0.03	0.98 ± 0.05	0.96 ± 0.05	0.95 ± 0.05
sBP vigil (mmHg)	135.4 ± 14.8	130.7 ± 18.9	136.2 ± 11.1	136.1 ± 10.5
dBP vigil (mmHg)	83.1 ± 7.8	81.1 ± 9.6	80.3 ± 8.0	79.2 ± 6.7
sBP sleep (mmHg)	123.1 ± 14.0	122.7 ± 18.8	125.2,3 ± 10.7	129.2 ± 14.9
dBP sleep (mmHg)	71.6 ± 8.5	70.7 ± 9.7	70.3 ± 6.8	71.3 ± 8.7
HbA1c (%)	8.0 ± 0.6	7.3 ± 0.8^€^	8.1 ± 0.7	7.3 ± 0.7^€^
Fasting glucose (mg/dL)	136.5 ± 30.0	125.6 ± 33.4	159.2 ± 38.0	111.6 ± 30.4^€^
Triglycerides (mg/dL)	139.1 ± 63.5	144.1 ± 66.2	152.6 ± 80.5	130.1 ± 85.3^€^
Total cholesterol (mg/dL)	171.7 ± 35.1	174.7 ± 33.3	186.8 ± 29.0	185.2 ± 42.0
LDL-cholesterol (mg/dL)	96.6 ± 32.0	101.9 ± 26.7	114.1 ± 21.0^§^	121.8 ± 25.6^£^
HDL-cholesterol (mg/dL)	46.2 ± 9.9	43.4 ± 8.3	42.0 ± 10.2	45.5 ± 11.8
Active GLP-1 (pmol/L)	5.0 ± 2.3	15.3 ± 10.3^€^	5.0 ± 2.4	5.6 ± 2.1^&^
C-reactive protein (mg/L)	2.5 ± 3.1	2.1 ± 2.4	2.7 ± 2.6	2.6 ± 1.6

Data are expressed in means ± SD. BMI, body mass index; GLP-1, glucagon-like peptide 1; sBP, systolic blood pressure; dBP, diastolic blood pressure. SITA, sitagliptin group; NPH, bedtime insulin NPH group; wk = weeks.

^€^ *p < 0.001* for the difference between before and after treatments.

^§^ *p = 0.019* for the difference between SITA and NPH groups before treatments.

^£^ *p = 0.019* for the difference between SITA and NPH groups after treatments.

^&^ *p* = 0.001 for the difference between SITA and NPH groups after treatments.

The dosages of metformin (2.4 ± 0.3 x 2.3 ± 0.6 mg/day; p = 0.46) and of glyburide (17.6 ± 3,1 x 18.1 ± 4.1 mg/day; p = 0.68) were similar in SITA and NPH groups respectively Also, 77.8% and 64.7% of sitagliptina and NPH groups were on statin therapy (p = 0.47) and 77.8% and 100% were on IECA or diuretics (p = 0.11), respectively. All medications were kept constant during the study.

Subjects were followed weekly for drug and dietary adjustments during the first 30 days and then monthly throughout the study period. At the time of entry, complete medical history, physical examination, anthropometric and laboratory evaluation were obtained. The study was approved by the Institutional Review Board-Comissão de Ética para Análise de Projetos de Pesquisa - *CAPPesq*. All participants gave written informed consent. The following procedures were performed before and after 24 weeks of treatment with either sitagliptin or bedtime NPH insulin:

### Laboratory determinations

Blood samples were collected for fasting plasma glucose (FPG), HbA1c, lipid profile, C-reactive protein and aGLP-1 levels. Glucose levels were determined by the glucose oxidase/peroxidase method (Labtest, Sao Paulo, Brazil) and glycated hemoglobin (HbA1c) by HPLC (National Glyco Hemoglobin Standardization Program, USA. Triglyceride levels were measured by the lipase/glycerol kinase method (Labtest, Sao Paulo, Brazil) and total cholesterol (total-C) by the cholesterol oxidase/peroxidase method. HDL-cholesterol (HDL-C) was separated using the phosphotungstic acid/Mg^2+^ method and measured by oxidase/peroxidase method. LDL-cholesterol (LDL-C) was estimated by Friedewald equation. Plasma active GLP-1 levels(aGLP-1) were measured using the ELISA Kit EGLP-35 (Millipore Corporation, Billerica, MA) on a Spectramax M5 fluorometer (Molecular Devices, Sunnyvale, CA). The inter assay and intra assay coefficients of variation were 6% and 4% respectively. C-reactive protein (CRP) was determined by high sensitivity ELISA kits (R&D Systems, Minneapolis, MN). All determinations were performed in duplicate.

### Cardiovascular evaluation

#### Blood pressure

Systolic and diastolic blood pressure (BP) values were assessed by 24-hour ambulatory BP monitoring in all patients every 20 min from 8.00 am to midnight, and every 30 min from midnight to 8.00 am in the following day.

#### Echocardiogram

Comprehensive 2-dimensional echocardiogram was performed in 15 patients of SITA group and 14 of NPH groups. They were positioned in a left lateral position, monitored with electrocardiogram to image acquisition from longitudinal, transversal, apical four, three and two chambers views, using either a Toshiba Artida (Japan) or a GE Vivid 7 (EUA) machine with 2,5 MHz transducers. Left atrium antero-posterior diameter, left ventricle diastolic and systolic diameters, septal and posterior wall thickness were measured. Left ventricular volumes were automatically calculated using Simpson’s rule from apical four and two chambers views. Measurements of mitral inflow included peak early diastolic velocity (E-wave), late diastolic velocity (A-wave) and E/A ratio by pulsed Doppler as well as basal septal early diastolic velocity (e’ wave) by tissue Doppler. Normal diastolic function was defined as follows: tissue Doppler septal e’ wave velocity ≥ 8 cm/s, mitral valve E/A velocities ratio > 1 and < 1.5, E/e’ ratio < 12 and, E wave deceleration time (DT) between 150 ms – 240 ms. Left ventricular diastolic dysfunction (LVDD) was defined as follows: e’ < 8 and classified as grade I: mild diastolic dysfunction (e’ < 8 cm/s, E/A < 0.8, E/e’ ≤ 8, DT > 200 ms), grade II: moderate diastolic dysfunction (e’ < 8 cm/s; E/A 0.8-1.5, E/e’ 9–12, DT 160 - 200 ms), grade III: severe diastolic dysfunction (e’ < 8 cm/s; E/A ≥ 2, E/e’ ≥ 13, DT < 160 ms) [12, 13].

The exams were analyzed by two experienced echocardiographers, blinded to medications. Six patients (3 SITA, 3 NPH) did not complete echocardiographic evaluation and were not included in this analysis.

### Statistical analysis

Data were analyzed using SPSS 15.0, SAS 8.0 and Excell 2003 and expressed as mean ± SD. For anthropometric data, the Student’s *t* test was used to compare the means. Differences in clinical characteristics and metabolic variables among groups were tested with two-way ANOVA models followed by Tukey’s multiple comparison tests. Fisher’s exact test was used to assess the association between the improvement in cardiac function and therapies. *P* ≤ 0.05 were considered statistically significant.

## Results

At baseline, there were no significant differences between the two groups with respect to gender, age, duration of diabetes, weight, BMI, waist-hip ratio, FPG, HbA1c, CRP, aGLP-1, total and HDL-cholesterol levels. LDL-cholesterol was lower in the SITA group (*p* = 0.019) (Table 1).

### Anthropometric and metabolic evaluation

Weight, waist/hip ratio and BMI did not change. Both treatments resulted in similar decrease in HbA1c values (*p* < 0.001). Bedtime NPH insulin therapy also reduced FPG and triglyceride levels (*p* < 0.001), but there was no difference in triglyceride levels between groups after treatment. CRP, total-C and HDL-C levels did not change after 24 weeks of either therapy and remained similar between groups, whereas final LDL-C levels were lower in the SITA group in comparison to the NPH group (*p* = 0.019), although this difference was already present at baseline. As expected, fasting plasma aGLP-1 levels increased three times following sitagliptin treatment (p < 0.001), and were higher than those following NPH treatment (*p* = 0.001) (Table 1).

### Cardiovascular evaluation

Systolic and diastolic ambulatory blood pressure did not change during periods of vigil or sleep with either treatment and were similar between groups (p > 0.05) (Table 1). No significant differences in the echocardiographic evaluation of the diastole were detected between groups at baseline. Left ventricular diastolic dysfunction (LVDD) was diagnosed in 53% (8/15) of patients in the SITA group and in 64% (9/14) of patients in the NPH group (*p* = 0.710) (Table 2).

**Table 2:** **Tissue and conventional Doppler Echocardiographic parameters and grade of left ventricular diastolic dysfunction by tissue Doppler echocardiograms at baseline and after 24 weeks of treatment with sitagliptin in 15 patients (SITA group) or bedtime NPH insulin in 14 patients (NPH group)**

Groups/Patients		Baseline				After	24-wk	
	e’	E/e’	E/A	LVDD	e’	E/e’	E/A	LVDD
**SITA** 1	7.0	10.1	0.9	II	7.0	2.9	0,5	I
2	7.0	5.1	0.6	I	11.0	4.7	0,6	0
3	8.0	9.5	0.7	0	10.0	5.7	0,6	0
4	7.0	7.6	0.9	I	9.9	9.8	1.1	0
5	6.0	9.7	1.8	II	9.1	8.2	2.0	0
6	7.4	7.2	1.1	I	7.0	6.3	0.8	I
7	8.0	8.1	1.0	0	9.0	10.0	1.0	0
8	8.0	4.3	0.6	0	6.8	5.9	0.6	I
9	10.0	6.3	0.8	0	8.0	8.4	1.5	0
10	15.0	6.0	0.8	0	7.0	10.4	0.7	I
11	7.0	11.1	0.9	II	8.0	8.8	1.0	0
12	10.0	5.6	0.7	0	8.0	8.1	0.8	0
13	4.7	12.1	0.8	II	6.1	6.6	0.5	I
14	10.0	6.1	0.9	0	6.4	7.5	0.7	I
15	7.0	10.0	1.1	I	5.8	12.8	0.9	I
**NPH** **1**	7.0	12.0	0.9	II	7.0	13.3	1.2	II
2	6.0	9.8	0.7	II	5.2	12.0	0.6	II
3	7.0	5.7	0.6	I	7.0	4.7	0.5	I
4	6.0	9.8	0.9	II	10.0	9.2	1.7	0
5	4.0	16.5	0.9	II	5.0	12.4	0.7	II
6	8.0	5.3	0.7	0	9.0	5.3	0.7	0
7	10.0	7.3	1.0	0	10.0	6.0	0.9	0
8	18.0	2.8	0.7	0	8.8	6.6	0.7	0
9	6.1	12.1	0.9	I	7.5	10.0	0.8	II
10	10.0	6.8	0.9	0	6.3	11.4	0.8	II
11	4.6	12.0	0.6	II	6.0	10.8	0.8	II
12	7.1	7.7	0.9	I	7.9	10.0	1.3	I
13	5.0	13.6	0.7	I	4,2	16.2	0.6	I
14	8.0	14.2	1.2	0	9.0	10.0	1.0	0

e’ = early diastolic velocity (cm/s); E/e’ = mitral inflow E velocity to tissue Doppler e’ ratio. E/A = early diastolic to late diastolic velocities ratio; LVDD = left ventricular diastolic dysfunction; 0 = absent LVDD; I = grade I LVDD; II = grade II LVDD; wk = weeks.

After 24 weeks of treatment, from the 8 patients with LVDD receiving sitagliptin, 2 patients showed LVDD improvement from diastolic dysfunction type II to type I and 4 patients moved from diastolic dysfunction type I to normal parameters (75%). On the other hand, from the 9 patients with LVDD receiving bedtime NPH insulin, 1 patient moved from diastolic dysfunction type I to normal parameters (11%). The difference in improvement rates between the two groups was significant (*p* = 0.015; OR = 24; CI = 1.74-331. Systolic function remained normal in all patients. Other parameters analyzed such as left atrium diameter, LV diastolic and systolic diameters and volumes and septal and posterior wall thickness were normal in all 29 patients evaluated and did not change significantly with treatments.

## Discussion

The main purpose of our study was to determine which of two anti-hyperglycemic agents, DPP-4 inhibitor or bedtime NPH insulin, would better address vascular dysfunction when used as add-on therapy to patients with T2D inadequately controlled with metformin plus glyburide. After 24 weeks of therapy, both agents yielded comparable effects concerning glucose control, as shown by similar HbA1c levels, evidencing that DPP-4 inhibitors can be used later in the course of the disease, still retaining effectiveness in patients with long-standing diabetes.

In addition, an improvement in LVDD was suggested in patients treated with the DPP-4 inhibitor in comparison to those treated with bedtime NPH insulin(p = 0.015).

As expected, a high proportion of our patients (59%) had LVDD [14] that was associated with preserved systolic function at baseline. It is well established that LVDD represents the earliest pre-clinical manifestation of diabetic cardiomyopathy, preceding systolic dysfunction [15, 16]. Early intervention, improving LVDD or preventing its onset, is extremely important because more than 30% of patients with normal LV systolic function but decreased diastolic function can progress to congestive heart failure [17].

Tissue Doppler echocardiogram markedly improved the detection of diastolic dysfunction in asymptomatic patients [14]. The clinical utility of myocardial velocity measurements for the assessment of diastolic function is widely accepted and has been documented previously [13].

It has been shown that hyperglycemia leads to LVDD and its correction can normalize the ventricular function of patients [18]. Nevertheless, in our study the DPP-4 inhibitor was more effective in improving ventricular dysfunction than bedtime NPH insulin despite similar glucose control for both and lower triglyceride level for NPH. That difference could not be attributed to baseline characteristics, as both treatment groups had equivalent prevalence of LVDD. Neither to chances in blood pressure, cholesterol levels and inflammatory status, attested by no changes on 24-hour ambulatory blood pressure, cholesterol and C-reactive protein levels. So, the increased aGLP-1 levels due to the DPP-4 inhibition in patients treated with sitagliptin might have contributed to the observed improvement in LVDD since beneficial cardioprotective effects of GLP-1 have been previously attested in non diabetic subjects [5, 6, 9].

Experimental data evidenced that sitagliptin may exert cardioprotective effects by increasing myocardial cAMP levels and cAMP response element-binding protein (CREB) phosphorylation [19], a nuclear transcription factor involved in ischemic preconditioning. Alternatively, sitagliptin may protect the stromal cell-derived factor-1α (SDF-1α), a substrate of DPP-4 that is overexpressed in response to tissue injury in the ischemic myocardium and is associated with increased number of circulating progenitor cells [20]. Sitagliptin administration stimulated resident cardiac stem cells and neovascularization and reduced cardiac remodeling, improving myocardial function and survival after myocardial infarction [21].

Anti-hypertrophic/fibrotic effects on cultured cardiac cells [8] and reduction of apoptosis and oxidative damage after ischemia/reperfusion, probably by activation of PI3K/Akt signaling pathway by GLP-1/GLP-1 receptor [22] collaborated to these results.

By inhibiting CD26 expression on mononuclear cells, sitagliptin could increase mononuclear cells homing to the infarct area and improve cardiac recovery and repair after acute myocardial infarction. The SDF-1alfa/CXCR4 axis plays a major role in cell homing to infarcted myocardium and is negatively regulated by CD226 [23].

Also, patients with heart failure had increased circulating levels of DPP-4 and there was an inverse correlation between serum DPP-4 activity and left ventricular ejection fraction [24].

Further effects include the reduction in renal sodium reabsorption, interactive hemodynamic effects involving angiotensin-converting enzyme inhibition and the increased level of substance P, that acts as vasodilator and is a substrate for DPP-4 [25].

### Other factors have to be considered

The possibility that the difference in LVDD after the intervention could be due to a deleterious effect of NPH rather than a beneficial effect of DPP-4 inhibitor merits attention. It is known that LVDD is associated with insulin resistance and a deleterious action of insulin on this parameter and on the development of cardiovascular disease has been suggested [26]. However, worsening of diastolic function associated with insulin therapy in our study was unlikely, as it was worsened in only 1 of 14 patients treated with insulin.

Also, considering the greater association of LVDD with impaired glucose tolerance but not impaired fasting glucose [27], a “probable” improved glucose control during wake time with sitagliptin could have influenced our results. Further, an improvement in insulin resistance, another great predictor of LVDD [28], could also have occurred.

Till now, there is no clear explanation for the increase in rate of hospitalization for heart failure observed with saxagliptin, another DPP-4 inhibitor [29]. This result does not agree with our data and with countless reports about the benefits of GLP-1 and DPP-4 inhibitors in cardiac function and merits further study.

No clinically meaningful differences were observed in the SITA group compared with NPH group with respect to body weight, BMI and waist-hip ratio, which remained unchanged in both groups after 24 weeks. Frequent dietary orientation during follow-up visits and the combined therapy with metformin in all of our patients might have prevented this. Overall assessment of safety demonstrated that both drugs were well tolerated in this study, and there were no significant side effects or severe hypoglycemic episodes.

### Limitations

Left ventricular diastolic function is one of the most difficult and controversial parameter to be evaluated in cardiology, considering the many clinical, demographic, hemodynamic, and echocardiographic variables. No criterion is assessed isolated to diagnose LVDD. For example, a reduced mitral E/A ratio in the presence of normal annular tissue Doppler velocities can be seen in volume-depleted normal subjects, so that an E/A ratio< 0.8 alone should not be used to infer the presence of diastolic dysfunction. Furthermore, Grade III LVDD should not be determinate by a single examination and requires serial studies after treatment is optimized. Echocardiographic cut-off values may have slight variations in different populations. We considered E/A ratio ranging from 1.8 to 1.9 with deceleration time between 150–240 ms as normal parameters. In the same way E/e’ ratio of 12.1 was considered as type 2 diastolic dysfunction.

We study a limited number of patients in both groups, so that further investigation with greater number of patients should be carried out.

## Conclusions

We report a similar effectiveness on glucose control of the DPP-4 inhibitor sitagliptin and bedtime NPH insulin as third-line agents in T2DM patients. Furthermore, the data in this cohort suggested that sitagliptin may have had cardioprotective effects, seemingly beyond and independent of glucose control, with positive effects on left ventricular diastolic function. Therefore, the DPP-4 inhibitor may be a promising drug for the prevention of diabetic cardiomyopathy.

## Abbreviations

BP: Blood pressure
BMI: Body mass index
CRP: C-reactive protein
DPP-4: Dipeptidyl peptidase-4
GLP-1: Glucagon-like peptide 1
HDL-C: HDL-cholesterol
LDL-C: LDL-cholesterol
LVDD: Left ventricular diastolic dysfunction
T2CM: Type 2 diabetes mellitus.
